# The Help for Hay Fever community pharmacy-based pilot randomised controlled trial for intermittent allergic rhinitis

**DOI:** 10.1038/s41533-020-0180-4

**Published:** 2020-06-01

**Authors:** Sarah Smith, Terry Porteous, Christine Bond, Jill Francis, Amanda J. Lee, Richard Lowrie, Graham Scotland, Aziz Sheikh, Mike Thomas, Sally Wyke, Lorraine Smith

**Affiliations:** 1 Institute of Applied Health Sciences, University of Aberdeen, Polwarth Building, Foresterhill, Aberdeen, AB25 2ZD UK; 2 School of Health Sciences, City, University of London, Northampton Square, London, EC1V 0HB UK; 3 Pharmacy Research and Development Team, Pharmacy and Prescribing Support Unit, West Glasgow Ambulatory Care Hospital, Glasgow, G3 8SJ UK; 4 Allergy and Respiratory Research Group, Centre for Medical Informatics, Usher Institute of Population Health Sciences and Informatics, The University of Edinburgh, Teviot Place, Edinburgh, EH8 9AG UK; 5 Primary Care and Population Sciences, University of Southampton, Aldermoor Health Centre, Aldermoor Close, Southampton, SO16 5ST UK; 6 Institute of Health and Wellbeing, College of Social Sciences, University of Glasgow, 27 Bute Gardens, Glasgow, G12 8RS UK; 7 Faculty of Pharmacy, Building A15, The University of Sydney, Camperdown, NSW 2006 Australia

**Keywords:** Respiratory signs and symptoms, Patient education

## Abstract

Management of intermittent allergic rhinitis (IAR) is suboptimal in the UK. An Australian community pharmacy-based intervention has been shown to help patients better self-manage their IAR. We conducted a pilot cluster RCT in 12 Scottish community pharmacies to assess transferability of the Australian intervention. Trained staff in intervention pharmacies delivered the intervention to eligible customers (*n* = 60). Non-intervention pharmacy participants (*n* = 65) received usual care. Outcome measures included effect size of change in the mini-Rhinoconjunctivitis Quality of Life Questionnaire (miniRQLQ) between baseline, 1-week and 6-week follow-up. Trial procedures were well received by pharmacy staff, and customer satisfaction with the intervention was high. The standardised effect size for miniRQLQ total score was −0.46 (95% CI, −1.05, 0.13) for all participants and −0.14 (95% CI,−0.86, 0.57) in the complete case analysis, suggesting a small overall treatment effect in the intervention group. A full-scale RCT is warranted to fully evaluate the effectiveness of this service.

## Introduction

Intermittent allergic rhinitis (IAR) (also known as seasonal AR or hay fever) is a major chronic respiratory disease with high UK prevalence, estimated at 26%^1^. Prevalence has increased substantially in recent years^2,3^. IAR has a significant negative impact on quality of life (QOL)^4^, productivity^5^, school performance^6^ and healthcare costs^7^. People with IAR are also at increased risk of suffering asthma, rhinosinusitis, and other related upper airway conditions^8,9^. Despite availability of evidence-based clinical guidelines for managing IAR in primary care, management of the condition in the UK is suboptimal^10–13^, suggesting significant avoidable morbidity.

Recent research in Australia has shown that a community pharmacist-delivered IAR intervention can improve patient outcomes^14–16^. Such an approach could potentially improve IAR management in the UK and other healthcare systems, as community pharmacists are well placed to recognise and recommend treatment for IAR, particularly for intermittent and mild cases^17–19^. However, it is unclear whether the IAR intervention will deliver the same benefits outside the Australian setting in which it was developed. Hence, locally conducted research is needed to inform policy and practice in other countries. Initially, estimates of effect sizes, participation rates and completion rates need to be derived to inform the design of a UK trial^20^. To our knowledge, no community pharmacy-based behaviour change intervention, designed to improve self-management of IAR, has been undertaken outside Australia.

The aim of this study was to conduct a pilot randomised controlled trial (RCT) in Scotland of ‘Help for Hay Fever’ (HFHF), a goal-focussed intervention for the self-management of intermittent IAR based on the Australian programme but adapted for the UK setting. Specific objectives were to test transferability of methods and measures of the intervention used in the Australian research and to inform the design of a future UK full-scale RCT. The research questions included: whether the pharmacy staff training programme, study materials and procedures were acceptable and suitable for use in a UK setting; whether the intervention was acceptable to pharmacy staff delivering and customers receiving the service; and whether the outcome measures were appropriate for quantification of pharmacy and customer recruitment and completion rates and for estimation of effect sizes and variability of change in allergic rhinitis-related QOL to inform a sample size calculation.

## Results

### Recruitment, retention and completion

**Fig. 1:**
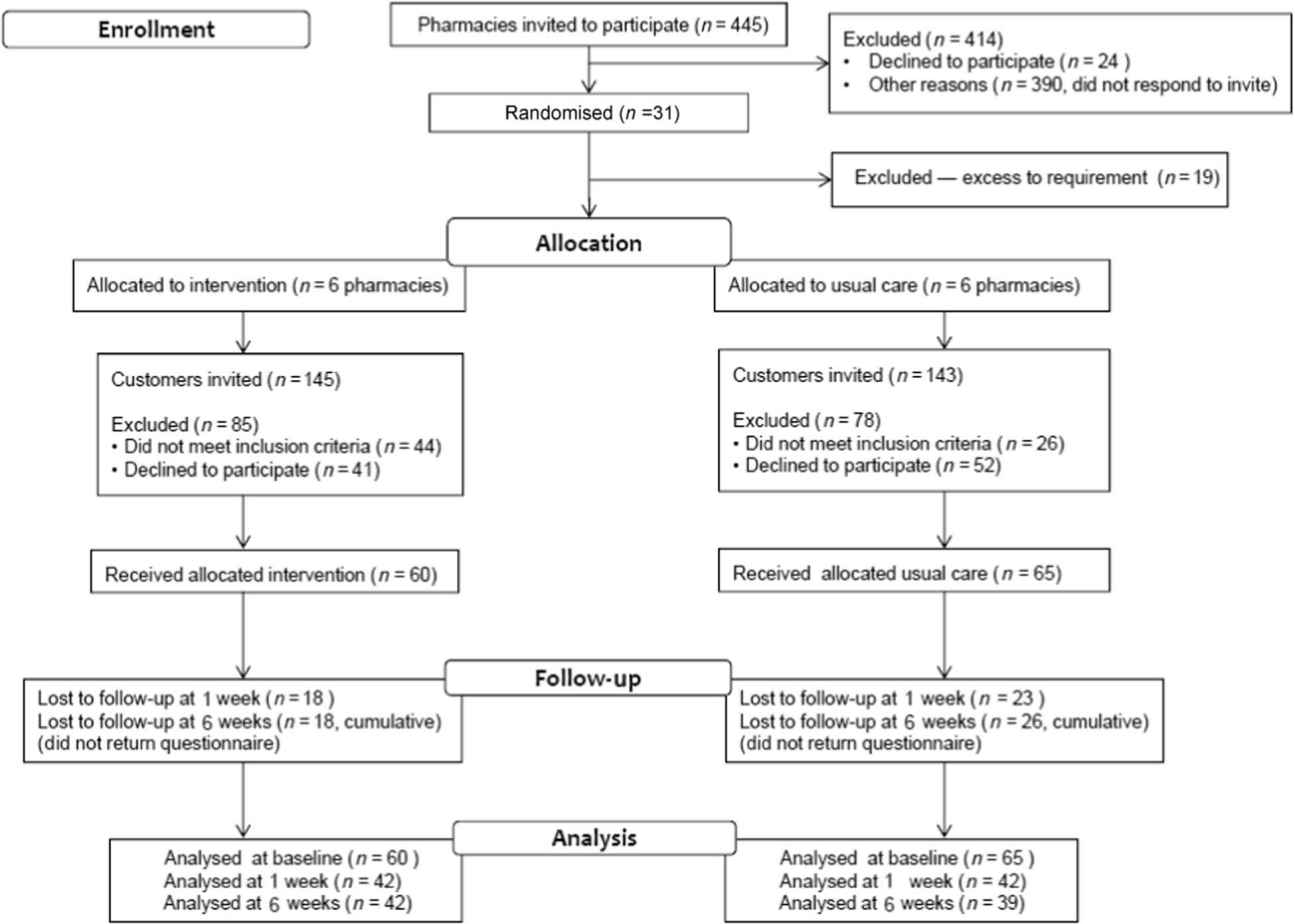
Flow of participants into the ‘Help for Hay Fever’ trial.

Figure 1 shows the flow of participants through the study.

Thirty-one pharmacies agreed to participate (7% of those approached, *n* = 31/445). Following randomisation, 12 pharmacies were recruited and retained, 6 in each arm, i.e. 3 intervention and 3 usual care pharmacies in each participating health board area (HBA) (Fig. 1).

Seven pharmacists and seven pharmacy assistants from the six intervention pharmacies (i.e. at least one member of staff from each pharmacy) attended the 3-h training event. Scores for the ‘before-and-after’ IAR knowledge test improved from 58% of questions being answered correctly before training to 85% following training. The evaluation form, completed by all attendees, showed training was very positively received. Content and format of the training, confidence of study procedures and general feedback achieved mean scores ranging from 8.2 to 9.3 out of 10. The lowest mean score (7.8/10) was for ‘role-playing’ of the intervention.

Completion of the daily recruitment log (recording numbers eligible, declined, recruited) was variable. Nonetheless, data from returned logs, whether fully or partially complete, showed that at least 288 customers were assessed for eligibility, and of these, 125 (43%) were recruited. Recruitment rates varied across pharmacies (Table 1).

**Table 1:** Recruitment by pharmacy.

Region							Total
Grampian							
Pharmacy ID	A056	A108	A125	A007	A014	A055	
Number of customers recruited	11	8	13	12	4	13	61
Greater Glasgow and Clyde							
Pharmacy ID	G054	G126	G269	G003	G210	G246	
Number of customers recruited	10	15	3	12	10	14	64
Total							125

Participants in both groups were mostly female and comparable in terms of employment status. Mean age was higher for the intervention group (43 years) than control (37 years), and Scottish Index of Multiple Deprivation (SIMD) scores (Supplementary Note 1)^21^ were lower in the intervention group (i.e. less deprived) (Table 2).

**Table 2:** Baseline patient characteristics.

Characteristic	Usual care (*N* = 65), *n* (%)	Intervention (*N* = 60), *n* (%)
Mean age (SD)	36.6 (16.2)	43.0 (15.9)
Male	30 (46.1)	25 (41.7)
SIMD^a^ (quintiles)		
Most deprived—1	18 (27.7)	10 (16.7)
2	18 (27.7)	4 (6.7)
3	9 (13.8)	4 (6.7)
4	8 (12.3)	12 (20.0)
Least deprived—5	12 (18.5)	30 (50.0)
Currently employed	49 (75.4)	45 (75.0)

*SD* standard deviation.

^a^SIMD—Scottish Index of Multiple Deprivation is a relative measure of deprivation^21^.

Retention rates for the intervention group at 1 week and 6 weeks were 70% at both timepoints and, for the control group, 65% and 60%, respectively (see Fig. 1). Only 71 customers (57%) returned questionnaires at all three timepoints.

### Quantitative outcome measures

IAR-related QOL values were calculated for both ‘total’ and ‘domain’ scores of the mini-Rhinoconjunctivitis Quality of Life Questionnaire (miniRQLQ)^22^. From baseline to 6 weeks, mean miniRQLQ total score decreased in both groups (usual care group by 1.00 (SD 1.34); intervention group by 1.45 (SD 1.26)), reflecting improved QOL for both groups but by slightly more in the intervention group (Table 3). A sensitivity analysis of only those respondents who provided complete miniRQLQ data at all three timepoints (i.e. complete case analysis) showed similar decreases (usual care group by 1.35 (SD 1.29); intervention group by 1.47 (SD 1.35)).

**Table 3:** Allergic rhinitis quality of life (MiniRQLQ Total Score^a^) by treatment arm across timepoints.

All participants	*n*	Usual care		*n*	Intervention	
		Mean	SD		Mean	SD
All participants						
Baseline	65	2.71	1.25	60	3.10	1.15
1 week	42	2.26	1.15	42	2.20	1.28
6 weeks	39	1.74	1.18	42	1.43	1.06
Change (W1–B)	42	−0.67	1.17	42	−0.74	1.17
Change (W6–B)	39	−1.00	1.34	42	−1.45	1.26
Complete case analysis						
Baseline	26	3.1	1.29	32	2.84	1.23
1 week	26	2.25	0.98	32	2.13	1.37
6 weeks	26	1.75	1.19	32	1.38	1.04
Change (W1–B)	26	−0.85	1.07	32	−0.72	1.15
Change (W6–B)	26	−1.35	1.29	32	−1.47	1.35

*W1* 1 week, *B* baseline, *W6* 6 weeks, *SD* standard deviation.

^a^MiniRQLQ mean total scores range from 0 to 6; lower scores reflect better quality of life.

Table 4 shows the main analysis of adjusted between-treatment difference in the 6-week mean miniRQLQ by domain and overall. The standardised effect size for miniRQLQ total score was −0.46 (95% confidence interval (CI), −1.05, 0.13) for all participants and −0.14 (95% CI, −0.86, 0.57) in the complete case analysis, suggesting a small overall treatment effect^23^ in the intervention group (Table 4). The effect size was, however, greater for the miniRQLQ domains of practical problems and nose symptoms. The intra-cluster correlation coefficients (ICCs) were close to zero for almost all miniRQLQ domains and the total score, indicating a non-significant effect of clustering by pharmacy (Table 4).

**Table 4:** Between-group differences in miniRQLQ scores and effect sizes.

MiniRQLQ domain	Between-group comparisons (intervention *N* = 60; usual care *N* = 65)					All participants (*N* = 125)		Complete case analysis (*N* = 58)	
	Difference between groups at 6 weeks	Estimated proportion with MCID^a^ change		Proportion benefitting from intervention	Number needed to treat	Effect size^b^ (95% CI)	ICC	Effect size^b^ (95% CI)	ICC
	Mean	Usual care	Intervention						
Activities	−0.40	0.35	0.50	0.15	6.7	−0.40 (−1.00, 0.19)	~0	0.08 (−0.62, 0.79)	~0
Practical problems	−0.76	0.34	0.57	0.23	4.4	−0.86 (−1.77, 0.05)	0.09	−0.47 (−1.58, 0.65)	0.11
Nose symptoms	−0.68	0.31	0.55	0.24	4.2	−0.68 (−1.51, 0.15)	~0	−0.56 (−1.63, 0.51)	0.11
Eye symptoms	−0.44	0.39	0.53	0.14	7.1	−0.44 (−1.21, 0.33)	~0	−0.16 (−1.07, 0.74)	~0
Other symptoms	−0.13	0.28	0.35	0.07	14.3	−0.13 (−0.73, 0.48)	~0	0.22 (−0.47, 0.92)	~0
Total score	−0.45	0.40	0.52	0.12	8.3	−0.46 (−1.05, 0.13)	~0	−0.14 (−0.86, 0.57)	0.03

*ICC* intra-cluster correlation coefficient.

^a^Minimal clinically important difference (MCID) is defined as a score change of at least 0.5.

^b^Effect size = between-group difference in mean score at 6 weeks adjusted for mean score at baseline and pharmacy clustering.

We found that some pharmacies struggled to recruit 12 customers, therefore, for the purpose of calculating a sample size for a future RCT, we would assume a conservative ICC of 0.01 and a cluster (pharmacy) size of four. A sample size of 240 participants per group would have 90% power to detect a difference in mean baseline to 6-week change in miniRQLQ of 0.4 (intervention −1.4 and usual care −1.0). This assumes a standard deviation of 1.31 and a 2-sided 5% significance level. Again, based on our data, we estimate a 40% attrition rate, so a total of 120 pharmacies (60 per arm) would each need to recruit seven customers to give an estimated total sample of 840.

It was of interest to examine the proportion of patients who showed a decrease in miniRQLQ of at least 0.5 scale points (minimally important within-group change) for each randomisation group. This proportion was 0.52 for the intervention group and 0.40 for the usual care group (a difference of 0.12 benefitting from the goal-focussed intervention and a number needed to treat of 8.3) (Table 4).

For symptom severity at 1 week (Spector scale)^24^, an improvement was observed in scores for nasal symptoms (but not for non-nasal symptoms) in both groups. For medication adherence at 1 week (Medication Adherence Rating Scale)^25^, there was improvement in both groups. Regarding self-efficacy at 1 week (adapted Lorig scale)^26^, scores improved slightly in the intervention group but not in the usual care group. Between-group differences were not statistically significant for any of these three measures.

### Economic analyses

The resource use and associated cost variables used in economic analysis are summarised by treatment allocation group in Table S1 (Supplementary Note 2). Based on the adjusted analysis, the intervention was associated with a non-significant increase in mean (95% CI) costs to the National Health Service (NHS) (+£2.55 (−£5.77 to £10.87) at 6 weeks, for a non-significant improvement in mean (95% CI) EQ-5D (EuroQoL 5-Dimensions) score (0.019 (−0.0438 to 0.0813)). However, the opposite direction of effect was observed on the EQ-5D at 1 week (−0.010 (95% CI, −0.080, 0.059); Table 5). Overall, mean (95% CI) costs to society (incorporating estimated patient costs and indirect productivity costs) were also non-significantly elevated at 6 weeks (+£7.00 (−£5.84 to £19.84)).

**Table 5:** Between-group differences on economic outcome measures.

Outcome	Usual care, mean (SD)	Intervention, mean (SD)	Mean difference (95% CI)^a^	ICC
Total NHS costs (*n* = 64)	£18.78 (17.67)	21.99 (15.30)	£2.55 (−£5.77, £10.87)	0.030
Societal costs^b^ (*n* = 56)	£23.72 (18.80)	31.25 (32.15)	£7.00 (−£5.84, £19.84)	~0
EQ-5D at 1 week (*n* = 83)	0.895 (0.181)	0.880 (0.156)	−0.010 (−0.080, 0.059)	0.11
EQ-5D at 6 weeks (*n* = 79)	0.900 (0.178)	0.920 (0.144)	0.019 (−0.044, 0.081)	~0

^a^Adjusted for pharmacy type and clustering and baseline EQ-5D.

^b^Incorporates total NHS, patient and indirect productivity costs.

### Qualitative analysis

One staff member was interviewed from each of the six intervention pharmacies (five pharmacists and one pharmacy assistant). Staff were asked to rate how difficult it was to recruit participants for the study; four out of six reported a ‘difficulty’ rating of ≥3 (scale range; 1–5). The time needed to complete recruitment was described as the main difficulty. One participant also commented that recruitment at their pharmacy was limited by a high number of immigrant customers who were unable to take part because of their limited English language. Completing the daily recruitment log during the study was frequently mentioned as a particular problem, mainly because pharmacy staff forgot to do it. Most staff said they were not aware of recruited customers having difficulty completing the questionnaire.

Delivering the intervention did not pose any problems for the majority of pharmacy staff. Half said that they would ‘definitely’ or ‘quite likely’ continue to deliver the HFHF service if intervention resources continued, while others mentioned that it would be dependent on funding. One participant said they would be unlikely to offer the service due to its time-consuming nature and small staff numbers. All pharmacies would want to be reimbursed for continuing to provide the service, and most thought payment of between £15 and £30 per customer would be required. This would potentially increase the cost of a future rollout beyond the cost of pharmacist time considered in the current evaluation (Table S1). Most also thought that offering the HFHF service from their pharmacy was beneficial for both customers and the pharmacy. Pharmacists valued the fact that pharmacy assistants, as well as pharmacists, were able to deliver the intervention service.

Semi-structured telephone interviews were conducted with 16 pharmacy intervention customers (seven male, nine female). When asked what the HFHF service had done for them personally, most responses referred to the advice they received on medication use. This included the benefits of trying a different type of medication, trying a medication that was symptom specific and medication adherence, for example:

It kick started you back into taking medication regularly. I had stopped doing it as it wasn’t convenient to get the medication… Once I started taking medication regularly I was feeling much better. It’s been revolutionary. (ID068, female, 40s).

She (the pharmacist) told me to try antihistamine and eye drops. I found these beneficial. You have no idea the difference this has made. The eye drops have helped enormously. (ID207, female, 60s).

Customers also mentioned that it had helped raise their personal awareness and increased their knowledge of how to manage symptoms of IAR, for example:

The questionnaire made me look at lots of symptoms that I presumed were caused by asthma. It helped me understand more about hay fever. (ID033, male, 30s).

Others mentioned the self-help aspect of the intervention, i.e. applying the personalised practical strategies they had formulated with the pharmacist/assistant to avoid/minimise hay fever triggers. These included: drying and washing indoors; changing clothes after being outside; closing windows when the pollen count was high; and taking medication before symptoms appeared. Most participants also felt the length of time spent with pharmacy staff was about right, although one person felt the pharmacist was rushed while another said ‘it was a bit long but worthwhile’.

No difficulties were identified with any of the study materials. Everyone said they would recommend the service to others with hay fever, and most would be ‘very willing’ to return for a similar service; only one participant was ‘somewhat willing’ saying there was no need for further help because his new medication worked.

Most participants would have been willing to pay up to £10 for the HFHF service they received. Those willing to pay ≥£15 usually had a high symptom severity score and/or were from a more affluent area. Six participants were not willing to pay as they either could not afford it or thought it a service the NHS should provide.

Suggestions on how the service could be improved in a future trial included: raising awareness that the service was available in their pharmacy, including through the use of social media; having uninterrupted time with the pharmacist; and allowing participants the option to complete data collection forms online to avoid the need of going to a post box.

## Discussion

We conducted the HFHF pilot RCT to assess the transferability of an Australian community pharmacy IAR intervention to a UK setting; the methods and measures used in the Australian research were tested with a view to informing the design of a future RCT. Our findings suggest that it is feasible to deliver HFHF in the UK. High participant satisfaction showed that the service was acceptable to customers and pharmacy staff alike; customers all reported benefits and pharmacists reported benefits for both the pharmacy and pharmacy staff. The methods employed were largely acceptable but some of the measures used appeared to lack sensitivity; future work should review other available measures. We noted that effect sizes were small, economic indicators equivocal and retention rates low. However, given the small scale of the study and the issues concerning the measures selected, a full trial informed by the findings of this pilot is warranted. While the cost analysis valued pharmacist time using standard unit costs, most pharmacists indicated that they would want to be reimbursed by £15–£30 per patient for providing the service. Inclusion of such a fee would need to be considered in future evaluations of cost-effectiveness. However, replacing pharmacy assistants for pharmacists would reduce the cost of providing the service.

Response from pharmacies to participate in the study was lower than expected; a 2001 study monitoring pharmacy customers’ hay fever symptoms recruited more than half of all pharmacies in Grampian (64/123)^12^. The low participation rate in this study may have been due, in part, to no invitation reminder letter being issued, as well as other pharmacy service targets taking priority (as indicated by some declining pharmacies). Previous researchers have reported that, although pharmacists recognise the importance of taking part in research, barriers such as lack of time and support, including financial support, can limit their capacity to participate^27^. This is consistent with our qualitative findings where reimbursement for any future provision of the HFHF service would be essential.

Study resources were insufficient to allow an investigation of the fidelity of the intervention as delivered and received. The fact that pharmacy staff were trained to deliver the intervention is no guarantee that the intervention was delivered as intended. Studies of fidelity show that, even after substantial training, adherence to an intervention protocol can be as low as 43% Hence, a future trial should also assess the potential for increasing the potency of the intervention by improving fidelity.

The time of year and weather were significant challenges for recruitment; delays in obtaining ethics approval meant recruitment started late in the hay fever season, and poor summer weather (lower temperatures and higher rainfall than usual) led to lower than usual pollen counts, resulting in lower than expected numbers of customers with problematic IAR symptoms. This meant that some pharmacies struggled to reach the recruitment target of 12 customers.

Although all customers (125) completed a baseline questionnaire at the pharmacy at recruitment, approximately one-third of the 1-week and 6-week questionnaires were not returned, despite multiple reminders. Low response rates when using self-completed postal questionnaires are not unusual in health services research and pose a significant threat to validity^28^; alternative methods for collecting follow-up measures may be warranted.

The minimal clinically important difference (MCID) for the miniRQLQ is defined as the smallest change in QOL score that is meaningful to the patient, but the actual value of the MCID can vary across studies. For example, in the current study, we used a value of 0.5 for the miniRQLQ, which was identical to the MCID for the RQLQ^29^. Juniper et al. suggested a slightly higher value of 0.7 for the miniRQLQ^22^, but a later paper by Barnes et al.^30^ disagreed with Juniper’s threshold. They pooled miniRQLQ MCIDs from nine placebo controlled RCTs of intermittent and persistent rhinitis and, using regression and meta-analytical approaches, estimated the MCID for the miniRQLQ to be 0.42, which is much closer to the one we used.

The necessarily small scale of this study had implications for some of our findings. There was a disparity in SIMD scores between the two groups at baseline, possibly reflecting the small numbers of participants. IAR-related QOL improved in both groups at 1 week, and at 6 weeks this was greater in the intervention group, albeit a modest difference; if the pilot study had commenced earlier in the hay fever season, this difference may have been greater. However, these results only indicate the likely effect sizes; the small numbers involved in this study provided insufficient power to assess statistical significance.

IAR-related QOL improved more in the usual care group than expected. This may be because of a potential Hawthorne effect^31^, a phenomenon in which study outcomes are thought to be influenced by the fact that participants are aware they are being observed, rather than by the intervention itself. In this case, the extra time the control group spent with pharmacy staff during the recruitment process and completing forms relating to their personal experience of IAR (all of which was over and above ‘usual care’) may have influenced outcomes. Also, keeping a daily record of symptom severity and medication adherence in the week after recruitment was a form of self-monitoring, which may have influenced behaviour^32^.

Some issues arose concerning the other measures used in this pilot study. Our measures of medication adherence^25^, self-efficacy^26^ and symptom severity^24^ revealed no significant differences between groups. This may have been due to small numbers of participants or may have been an artefact caused by a Hawthorne effect. Also, the first two of these measures are not specifically validated for IAR, which may account for their lack of sensitivity. Similarly, our results suggest that the EQ-5D may not be sufficiently sensitive to detect meaningful between-group differences in IAR-related QOL.

Here we compare our findings to those of the Australian research^15,16^. It should be noted, however, that, unlike Smith et al.^15^, the current study was a pilot with insufficient statistical power to detect differences.

AR-related QOL improved significantly in the Australian studies^15,16^. The total score for the miniRQLQ mean difference between groups exceeded the MCID of 0.5 in both studies^15,16^. In the first study^16^, symptom severity also improved significantly. This study^16^ showed that participant-reported symptom severity improved significantly in patients whose goals and strategies were set by trained pharmacists, compared to patients who set their own goals and strategies. Self-efficacy was also found to improve significantly both within and between groups in the second study^15^.

Medication adherence was not found to be a significant factor in either of the Australian studies in terms of behaviour change and IAR self-management^15,16^. Although adherence improved in both groups after 1 week in the current study, there were no significant differences between groups in change scores. However, interestingly, adherence was also found not to be a significant predictor of symptom severity^15^, whereas AR-related QOL and strategies to control AR-related triggers was a significant predictor.

We anticipate that the low pharmacy response rate might be improved by: issuing reminder letters, promoting the trial via local pharmacy groups to further encourage involvement, reminding pharmacists that the training would be continued professional development for their portfolio, linking the service to existing extended services such as the Chronic Medication Service (Scotland) or the Medicines Use Review (rest of UK), providing appropriate incentives, and digitally augmenting the intervention through text messaging and/or a dedicated Facebook page. We might also increase the number of pharmacy assistants trained to deliver the intervention to reduce demands on pharmacists’ time.

To maximise the pool of potential customer participants, customer recruitment in any future trial should be conducted at the start of the hay fever season. In addition, to speed up recruitment, pharmacies would be required to achieve a certain percentage of the recruitment within a specific time period or otherwise be replaced by another pharmacy. In this way, we would hope to ameliorate the effects of community pharmacies achieving particularly slow recruitment rates. One disadvantage of this approach would be that more pharmacies would need to be trained as ‘standbys’. However, it is anticipated that staff (and ultimately the pharmacy) would benefit from the training, even if they did not participate in the research.

A future trial should place more emphasis on the importance of returning questionnaires and offer a variety of formats for completion (e.g. by telephone or electronically), as was suggested in customer interviews. Edwards^28^ has noted that financial incentives to return questionnaires can increase response rates. In future, the gift voucher given to customer participants to thank them for taking part may be better delivered on receipt of the final questionnaire, rather than at recruitment, which might also improve retention rates.

If the larger-than-expected improvement in outcomes in the ‘usual care’ group was in fact the result of a Hawthorne effect, it would be important to minimise the level of interaction between pharmacy staff and study participants. This is a challenging aspect of the current study design because the processes for recruiting participants and collecting data necessarily require such interactions. One solution might be to use a Solomon’s design, which recruits a third group of participants and gives them no measures to complete except the final primary outcome measures^33^.

The lack of a clinically important difference in the EQ-5D score between groups in this pilot suggests that it may not be sensitive enough to capture the value of improvements in IAR symptoms. However, failure to detect an important difference may simply reflect the small numbers and/or unexpected improvements in the control arm of the trial. While use of the EQ-5D (or another similar but potentially more sensitive generic instrument) should be retained in any future trial, a future economic evaluation could also include some primary preference elicitation work to assess the value that patients or the public place on IAR-specific improvements in health-related QOL (HRQOL).

Our findings suggest that the HFHF service, delivered in Scottish community pharmacies, is feasible to deliver and acceptable to customers and pharmacy staff and may have the potential to improve IAR management. This pilot study has derived data on estimates of effect sizes, participation rates and completion rates, which can be used to inform a future RCT. Based on these data, we have proposed modifications to pharmacy and customer recruitment procedures, measurement procedures and instruments and have suggested ways to maximise retention rates.

The economic analysis undertaken in this pilot work suggests that the intervention may increase costs to the NHS, particularly if it requires a fee for service, and may result in a small improvement in general HRQOL at 6 weeks. Longer-term follow-up in a definitive trial is required to ascertain whether any differences are real and sustainable. Cost-effectiveness of a future trial from an NHS perspective will likely depend on the price at which pharmacists are willing to supply the service and the extent to which pharmacy assistants can substitute for pharmacists, as well as the clinical significance and durability of any observed improvements in HRQOL. From a societal perspective, costs may also be offset by potential improvements in workplace performance.

A full RCT is now needed to determine whether the clinical benefits suggested by this pilot can be replicated, or even enhanced, on a larger scale. If findings from the full trial indicate that the HFHF intervention is a cost-effective way to help individuals better manage their seasonal IAR, this goal-focussed, behaviour change intervention could be rolled out to all community pharmacies.

## Methods

The protocol for this pilot trial is published elsewhere^34^. A summary of methods is presented here. Changes to the original protocol are listed in Supplementary Note 3.

### Study design

This was a community pharmacy-based pilot cluster RCT of a goal-focussed self-management intervention for IAR with associated economic analysis and a qualitative evaluation of the acceptability, experiences of and satisfaction with the intervention and acceptability of the measures used. The trial was conducted in two Scottish HBAs: (1) Grampian and (2) Greater Glasgow and Clyde. Recruited pharmacies (clusters) were randomised to deliver the HFHF intervention or to deliver ‘usual care’ to customers with IAR. Trained pharmacy staff recruited and consented eligible pharmacy customers using pre-specified inclusion/exclusion criteria (see below). Ethical approvals were obtained from the North of Scotland committee of the National Research Ethics Service (NRES) in May 2012, prior to commencing the research.

### Recruitment

Our target was to recruit 12 community pharmacies, 6 in each HBA. All community pharmacies in both HBAs (*n* = 445) were invited by letter to participate in the study. No reminders were sent. Consenting pharmacies were stratified by location and by pharmacy status (independent/small multiple/national multiple) and were randomised to intervention or usual care^34^. Pharmacies that consented but not included were informed of this by letter and asked if they would be willing to be on ‘stand by’ should any of the selected pharmacies drop out.

Pharmacy staff in both intervention and usual care groups received training in recruitment and written informed consent of study customers. Staff from intervention pharmacies attended a 3-h training event about IAR, its management^17^ and delivery of the intervention^34^; this included ‘role play’ where attendees worked in pairs to practise receiving and delivering the intervention.

Knowledge of IAR and its management was assessed before and straight after training using a knowledge-based multiple-choice questionnaire. For the purpose of informing training in a future RCT, staff satisfaction with, and acceptability of, the training were evaluated immediately after the event using a questionnaire adapted from one used previously in the Australian study^35^.

Customer recruitment began at the end of June 2012 for 4 months. In all pharmacies, potential participants were identified when they asked for advice about IAR symptoms, or requested medication for IAR, or presented a prescription for IAR medication. All willing participants completed an eligibility checklist before recruitment. Inclusion criteria were: being >18 years of age; having a history of IAR; having active symptoms of intermittent IAR as defined by ARIA (Allergic Rhinitis and its Impact on Asthma) guidelines^36^; being able to speak, write and understand English. Exclusion criteria were: being pregnant; having a terminal illness; having symptoms not suggestive of IAR; having taken part in an allergic rhinitis study within the past 2 years. Each pharmacy was asked to record daily recruitment data, thus providing a number log of customers who were ineligible, recruited or declined to participate.

### The HFHF intervention

The intervention was based on an Australian study^14–16^. To enhance its replicability, components of the intervention were specified^37^ using a validated taxonomy of behaviour change techniques^38^. Eligible customers received the intervention, delivered in one session by intervention pharmacy staff^34^. Customers were counselled on IAR management and treatment and provided with a card containing two pre-determined goals:

- to avoid/minimise hay fever triggers
- to eliminate/minimise hay fever symptoms.

In this card, participants wrote down their personal IAR triggers and symptoms, and pharmacy staff supported them in formulating personalised action plans for achieving the two goals. The purpose of this card was to engage customers in the self-management of their IAR; personally selected health-related goals are more likely to lead to positive changes in illness-related behaviour^39^. Participants also received printed information about IAR symptoms, triggers and other general IAR information. Customers from non-intervention pharmacies received usual care.

### Data collection

The baseline questionnaire was used to collect data about participants’ current experience of IAR including: IAR-related QOL (validated miniRQLQ—see Supplementary Note 4)^22^, generic health status (EQ-5D)^40^, symptom severity^24^, Work Productivity and Activity Impairment (WPAI)^41^, medication adherence^25^, and self-efficacy with respect to IAR management^26^. Participating customers in both groups were given a record card to complete daily in the week following recruitment; on this card they recorded a symptom severity score and adherence to IAR medication. All participants were mailed a second questionnaire (identical to baseline) 1 week after recruitment, and 6 weeks after recruitment they were mailed a third questionnaire to collect some outcome data (miniRQLQ, EQ-5D, WPAI)^34^ and additional data for economic analysis of any future RCT. Participants who had not returned their 1- or 6-week questionnaire 2 weeks after they were sent out received a reminder by telephone or email^34^. The 6-week questionnaire was sent to participants even though they did not return their 1-week questionnaire.

Semi-structured interviews with intervention pharmacists and a purposive sample of intervention customers (selected to represent a range of gender, age and symptom severity) were conducted to elicit views on experiences of and satisfaction with the intervention and the trial methods. Interview schedules were informed by the findings of the Australian research^35^.

### Outcome measures

The outcome measures were: community pharmacy and pharmacy customer recruitment; retention and completion rates; effect size and variability of change in IAR-related QOL (assessed by the miniRQLQ) between baseline, 1 week and 6 weeks. The miniRQLQ comprises four domains with three items and one with two items measuring, on a 0–6 response scale, the impact of IAR symptoms on QOL: activities, practical problems, nose symptoms, eye symptoms, and other symptoms. Mean scores are calculated for each domain and a total score for all 14 items. We considered a decrease of 0.5 in mean total score to be a clinically relevant improvement.

Other outcomes were: symptom severity, medication adherence and self-efficacy (i.e. belief in one’s ability to succeed in specific situations or accomplish a task)^42^ and acceptability of and satisfaction with study components (community pharmacy staff and pharmacy customers).

Pharmacy consultation time was costed for all participants using a published cost per-hour of community pharmacist time^43^. Medications either prescribed by pharmacists or obtained through the Minor Ailments Scheme at the initial consultation were valued using the British National Formulary (BNF) list price^44^, while those purchased over-the-counter were costed at their retail price. Data on use of health services over the follow-up period were estimated from patient questionnaires administered 6 weeks post-randomisation, as was overall use of prescribed and over-the-counter medications (type and quantity). Health service consultations were costed using unit costs derived from routine sources^43^, and medications were costed using BNF list prices or purchase prices as appropriate. The WPAI questionnaire was tested 1 week post-randomisation and any reported time lost from work was valued using age/sex-specific average gross wage rates^45^. Total NHS costs and broader societal costs (incorporating patient out-of-pocket medication costs and indirect costs of lost production) were then estimated per patient based on the components described above.

### Sample size

As this was a pilot RCT, no formal sample size calculation was undertaken^46^; however, sufficient numbers were required to estimate effect sizes and give a reliable ICC to inform a future full evaluation. We aimed to retain 10 pharmacies (5 per study arm) with 10 patients in each pharmacy. Therefore, to allow for attrition, our initial target recruitment was 12 pharmacies each recruiting 12 customers (i.e. total sample size of 144 patients).

### Data management and analysis

Data were entered into SPSS (version 20) and analysed using Stata version 14 (StataCorp LP, College Station, TX, USA). For descriptive analysis, mean (SD) miniRQLQ total scores across each timepoint were summarised by randomisation group for all participants and for complete cases (as a sensitivity analysis). A multi-level, mixed-effect linear regression model was applied using the ‘xtmixed’ command to fit linear mixed models for the miniRQLQ and its subscales. The model was used to determine the treatment effect (i.e. the difference in 6-week miniRQLQ score between the intervention and usual care group, adjusted for clustering by pharmacy). We reported results based on all participants as well as a complete case sensitivity analysis. It should be noted, however, that, since this was a pilot study, it is recognised that formal statistical analysis is underpowered and effects should be interpreted as descriptive.

Analysis of the economic outcomes adopted the same approach as used for the miniRQLQ and focussed on estimating differences in costs to the NHS (incorporating intervention and follow-up costs) and costs to society (incorporating patient and indirect costs) at 6 weeks, and differences in EQ-5D score at 1 week and 6 weeks (adjusted for clustering, pharmacy type and baseline EQ-5D score). Given the small numbers and short-term nature of this exploratory pilot study, we do not report a full incremental cost-effectiveness analysis.

One researcher (S.S.) carried out thematic descriptive analysis of the qualitative data. Interpretation and allocation of themes were discussed and cross-checked by a co-author (L.S.) to minimise potential researcher bias.

This pilot cluster RCT has followed the CONSORT 2010 checklist of information to include when reporting a pilot or feasibility trial (Supplementary Note 5).

### Reporting summary

Further information on research design is available in the Nature Research Reporting Summary linked to this article.

## Supplementary information

Reporting Summary

Supplementary Information

## Data Availability

The data that support the findings of this study are available from the corresponding author upon reasonable request.
