# Supplementary material for: The Help for Hay Fever community pharmacy-based pilot randomised controlled trial for intermittent allergic rhinitis
Source: NPJ Prim Care Respir Med. 2020 Jun 1;30:23. doi: 10.1038/s41533-020-0180-4 (PMC7264195; doi:10.1038/s41533-020-0180-4)
Supplement: Supplementary file 2 — Supplementary Information [file 41533_2020_180_MOESM2_ESM.pdf]

Supplementary Information File

Supplementary Note 1

Scottish Index of Multiple Deprivation (SIMD)

The SIMD is used by the Scottish Government to identify small area concentrations of multiple deprivation across all of Scotland in a consistent way. It combines 38 indicators across 7 domains, namely: income, employment, health, education, skills and training, housing, geographic access and crime. Further information about SIMD can be found on the Scottish Government website: (https://www2.gov.scot/Topics/Statistics/SIMD)

Supplementary Note 2

Detailed economic analysis

Aim

The purpose of economic analysis was to help determine the most appropriate health economic outcome measures for use in a future definitive evaluation.

Methods

Instruments were developed to collect data on pharmacist time, medications prescribed and purchased (at the initial consultation), and patients use of health services and prescribed and purchased medications over the 6-week follow-up period. The impact on generic health related QOL was assessed using the EQ-5D,[1] and productivity was measured using the allergy specific 6-item Work Productivity and Activity Impairment Questionnaire.[2] Finally, ex-post willingness to pay was assessed in a sub-sample of intervention recipients, and willingness to accept (supply) was estimated for a sub-sample of pharmacists.

Pharmacy consultation time (stripped of research time) was initially costed for all participants in both arms of the trial, using a published cost per hour of community pharmacist time.[3] Following this, based on pharmacists' willingness to supply the service, it was assumed that from an NHS perspective the active intervention would require a minimum fee for service payment of ~£10 per patient. Medications prescribed by pharmacists at the initial consultation were valued using the BNF list price.[4] while those purchased over the counter were costed at their retail price.

Data on use of health services over the follow-up period were estimated from patient questionnaires administered at 6 weeks post randomisation, as was overall use of prescribed and over the counter medications (type and quantity). Health service consultations were costed using unit costs derived from routine sources,[3] and medications were costed using BNF list prices[4] or purchase prices as appropriate. The EQ-5D was collected at baseline, 1 week and 6 weeks, and was scored using the UK population-based time-trade-off tariff,[5] providing a preference-based utility weight for the health status of each patient at each timepoint. The Productivity questionnaire was tested at 1-week post randomisation and any reported time lost from work was valued using age/sex specific average gross wage rates.[6]

Analyses were conducted from both an NHS and broader societal perspective (incorporating patient costs and indirect costs of lost production). From the NHS perspective, it was assumed that the NHS would incur the full costs of increased pharmacist time or any fee for service associated with the intervention.

Individual resource use and cost variables were summarised by treatment allocation group. Individual cost variables (time, medications, health service consultations) were then totalled for each individual patient (generating total cost variable) and the total QALYs for each patient were derived from the EQ-5D responses. The impact of the intervention on costs and QALYs was assessed using a mixed effects model, with a random-effect for pharmacy to adjust for clustering of participants within this unit of randomisation. Uncertainty surrounding the joint estimates of incremental costs and effects was assessed using non-parametric bootstrapping, and the impact of altering costing assumptions (for pharmacy consultations) was assessed using deterministic sensitivity analysis.

Results

The resource use and cost variables used for economic analysis are summarised by treatment allocation group in Supplementary Table 1. The mean reported time taken to consult patients, and the associated costs, were higher in the intervention group. The costs of drugs supplied at the initial consultation were somewhat lower in the intervention group, both prescribed and over the counter.

Supplementary Table 1. Preliminary descriptive statistics for the variables to be used in the economic analysis

	Usual care			Intervention			Cluster adjusted difference (95% CI)
**Variable**	**N**	**Mean**	**SD**	**N**	**Mean**	**SD**	
Pharmacist records							
Pharmacy time (hours)	64	0.16	0.11	51	0.23	0.14	0.072 (-0.018 to 0.163)
pharmacy costs (£) *	64	8.38	5.66	51	12.18	7.53	3.83 (-0.95 to 8.62)
NHS drug costs (£ BNF)	65	£1.63	£4.77	59	£0.58	£1.47	-1.46 (-3.66 to 0 .75)
OTC drug costs (£)	65	£2.47	£3.78	59	£0.68	£1.64	-1.75 (-3.11 to -0.37)*
NHS service use (and costs) to 6 weeks ++							
GP visits	39	0.128	0.409	42	0.095	0.297	-
GP nurse visits	39	0	0	42	0.024	0.154	-
Pharmacy visits	39	0.410	0.993	42	0.333	0.650	-
A&E attendances	39	0	0	42	0	0	-
NHS24 calls	39	0	0	42	0	0	-
Hospital days	39	0	0	42	0	0	-
NHS service use costs	39	£7.31	£15.14	42	£5.74	£11.67	-£1.26 (-8.59 to 6.08)
Medication costs (to 6 weeks) from patient questionnaires							
To patients OTC	36	£3.53	£5.02	37	£2.49	£3.52	-1.04 (-2.99 to 0.92)
To the NHS (prescribed or MAS)	36	£4.38	£7.96	37	£4.60	£6.78	0.22 (-3.13 to 3.56)
Health related Quality of life							
EQ_5D_basline (index score)	65	0.877	0.210	59	0.884	0.126	-
EQ_5D_1 week (index score)	42	0.895	0.181	41	0.880	0.156	-
EQ_5D_6 weeks (index score)	38	0.900	0.178	41	0.920	0.144	-
Quality adjusted life years (QALY)	33	0.102	0.019	35	0.104	0.016	0.0018 (-0.0079 to 0.0114)
Value of time lost from work							
Baseline	65	£4.47	£28.99	60	£1.96	£15.21	-
Week 1	42	£0.00	£0.00	41	£0.64	£4.09	-

Notes: * Pharmacist consultation time valued using the published unit cost for community pharmacist time (PSSRU); ++ NHS consultations costed using unit costs derived from published estimates (PSSRU); Prescribed medicines or medicines received through the minor ailments service valued at the BNF list price.[4]

No clear differences were observed between patients in the intervention and usual care group in relation to use of NHS services over the follow-up period or use of medications (both prescribed and over the counter). No patients, either in intervention group or usual care group, reported using NHS 24, A&E or other hospital services in relation to their AR.

The EQ-5D showed some evidence of improvement in both groups over the follow-up period, but the unadjusted difference in QALYs between groups was very small.

Incremental cost-effectiveness

While the numbers of participants with complete costs and EQ-5D data were small, multilevel models were tested as a means of estimating the incremental costs and QALYs associated with the intervention, allowing for clustering of observations within pharmacies. Models were also adjusted for the minimisation variable, type of pharmacy (independent, National chain), and baseline health status (EQ-5D). Supplementary Table 2 provides the results of these analyses using all the available data for each outcome. Supplementary Table 3 provides an analysis of EQ-5D scores and derived QALYs using only those participants responding at all three timepoints. These analyses are indicative of a tendency for increased costs in the intervention group. The intervention is associated with slight decrease in the EQ-5D score at 1 week, but a slight increase at 6 weeks.

Incremental cost effectiveness ratios (ICERs) were estimated based on an analysis of all patients with complete cost and QALY data (Supplementary Table 4). While the results are indicative of increased costs associated with the active intervention (particularly under fee for service assumptions), the estimated QALY differences over the 6-week follow-up are negligible.

Willingness to pay and willingness to accept

From the small subsample of 16 patients who were asked to provide their ex post maximum willingness to pay for the service, the estimated median value (range) was £12.50 (£0-£40). Of the six pharmacists asked their minimum willingness to accept (per patient to supply the service), the median value was £10 (£5-£15).

Supplementary Table 2. Summary of output form multilevel models for costs and EQ-5D

	Total NHS costs (n=64)	Societal costs (n=56)	EQ-5D 1 week (n=83)	EQ-5D 6 weeks (n=79)
Constant	£44.98 (£23.18 to £66.79)	£66.66 (£31.67 to £101.64)	0.1980 (0.0479 to 0.3482)	0.4579 (0.2742 to 0.6417)
**Intervention effect**	**£2.55 (-£5.77 to £10.87)**	**£7.00 (-£5.84 to £19.84)**	**-0.0104 (-0.0797 to 0.0589)**	**0.0188 (-0.0438 to 0.0813)**
Pharmacy type	-£3.34 (-£12.92 to £6.24)	-£6.68 (-£20.97 to £7.60)	-0.0066 (-0.0812 to 0.0680)	-0.0114 (-0.0833 to 0.0605)
EQ-5D (baseline)	-£28.56 (-£52.25 to -£4.87)	-£46.45 (-£85.35 to -£7.55)	0.7993 (0.6406 to 0.9581)	0.5048 (0.3041 to 0.7055)
ICC	0.030	~0	0.11	~0

Supplementary Table 3. Summary of output from multilevel models for complete case EQ-5D responses and derived QALYs

	**EQ-5D 1 week (n=68)**	**EQ-5D 6 weeks (n=68)**	**Total QALYs (n=68)**
Constant	0.1090 (-0.0422 to 0.2602)	0.4577 (0.2596 to 0.6557)	0.0286 (0.0136 to 0.0435)
**Intervention effect**	**-0.0254 (-0.0800 to 0.0292)**	**0.0209 (-0.0506 to 0.0924)**	**-0.0004 (-0.0060 to 0.0053)**
Pharmacy type	0.0028 (-0.0588 to 0.0645)	-0.0047 (-0.0855 to 0.0761)	0.0002 (-0.0062 to 0.0065)
EQ-5D (baseline)	0.8934 (0.7261 to 1.0608)	0.4995 (0.2803 to 0.7187)	0.0849 (0.0683 to 0.1014)
ICC	~0	~0	0.016

Supplementary Table 4. Summary of the change in costs and QALYs, and the incremental cost per QALY using patients with complete cost and QALY data

**Perspective/scenario**	**Difference in cost (£)**	**Difference in effect (QALYs)**	**Incremental cost per QALY gained**
NHS (base case); n = 54	£2.54 (-£6.32 to £11.40)	-0.0018 (-0.0079 to 0.0043)	Dominated
Societal (base case); n = 53	£6.93 (-£6.76 to £20.62)	-0.0015 (-0.0077 to 0.0047)	Dominated
**Sensitivity analyses**			
NHS (pharmacy intervention charge: £10 per patient); n = 60	£8.77 (£0.63 to £16.91)	-0.0002 (-0.0059 to 0.0055)	Dominated
NHS (pharmacy intervention charge: £20 per patient); n = 60	£18.77 (£10.63 to £26.91)	-0.0002 (-0.0059 to 0.0055)	Dominated

Discussion points

In relation to the assessment of methods for estimating cost-effectiveness, the economic instruments and questions were generally well tolerated, with similar response and completion rates to those of the clinical outcome measures. Several lessons can be taken forward to inform the design of a future trial based economic evaluation:

1) While the pharmacists were asked to factor research time out of their estimates of time taken to counsel/advise participants on their AR, time estimates for the usual care group suggest that time taken to recruit patients may have been counted by some pharmacists. It may be worthwhile observing a sample of consultations in the usual care and intervention arm of any future definitive trial to get a more objective handle on the time and nature of the consultations.
2) Medication collection methods generally performed well, although the tabular system devised for recording types, brands, quantities, and sources of medications can be improved.
3) No patients in this sample reported using any hospital-based services in relation to their AR during the study period, suggesting that these questions can be omitted from future resource use questionnaires.
4) While the EQ-5D scores showed some improvement in both arms at 6 weeks, the results are suggestive of a slightly greater increase in the active intervention (Supplementary Table 2). However, the 1-week results are suggestive of the opposite, with the intervention associated with a utility decrement at this timepoint. Consequently, there are negligible differences in QALYs between the two arms over the 6-week follow-up period. Further, the

observed utility gain at 6 weeks is below the previously identified mean minimally important difference for the EQ-5D (0.074).[7] While use of the EQ-5D or another similar but potentially more sensitive instrument (e.g. the SF-12 scored via the SF-6D) should be retained in any future trial, some primary valuation work to establish the value of any improvements in disease specific outcome measures may also be advisable.

5) The pilot analysis was based solely on the observed data within a 6-week follow-up period. However, any health benefits of the service resulting from behaviour change might in theory be maintained (or partially maintained) throughout the entire hay fever season and beyond. It would be prudent to include a longer-term follow-up of health-related QOL in any future trial, and to use extrapolation modelling to determine cost-effectiveness under alternative assumptions about maintenance of behaviour and QOL changes.
6) Finally, the pilot WTP and WTA questions suggested that participant ex-post WTP might exceed pharmacists minimum WTA, suggesting that the service might be cost-beneficial from a private payer's perspective. However, such an approach would need to be refined and preferably based on ex ante WTP values if a decision were to be made to adopt a cost-benefit analysis framework in a future economic evaluation. From a private payer's perspective, WTP values would preferably reflect inexperienced sufferers' willingness to pay for the demonstrated health benefits of the intervention. To inform an NHS perspective on provision, WTP values should preferably reflect general population preferences.

<u>Supplementary Note 3</u>

Changes from the original protocol[8]

Timetable: Delays in finalising the funding and receiving full approval from NRES meant resulted in a corresponding delay in starting customer recruitment. To compensate, customer recruitment was extended until the end of October 2012.

Stratification of the sample: The protocol stated that the sample would be stratified by location (Grampian or Greater Glasgow & Clyde), pharmacy status (independent/ multiple), and number of FTE staff. Instead, stratification was by location and pharmacy status only; insufficient pharmacies responded to allow stratification by number of FTE staff.

<u>Supplementary Note 4</u>

Mini Rhinoconjunctivitis Quality of Life Questionnaire (miniRQLQ©)

Respondents are asked to indicate on a scale of 0 (not troubled) to 6 (extremely troubled), how they have been affected during the previous week by nose/eye symptoms associated with rhinoconjunctivitis. Symptoms in 5 domains are assessed:

Activities:

- Regular activities at work and at home (e.g. housework, gardening)
- Recreational activities: (e.g. sports, social activities, hobbies)
- Sleep

Practical problems:

- Need to rub nose/eyes
- Need to blow nose repeatedly

Nose symtoms:

- Sneezing

- Stuffy blocked nose
- Runny nose

Eye symptoms:

- Itchy eyes
- Sore eyes
- Watery eyes

Other symptoms:

- Tiredness and/or fatigue
- Thirst
- Feeling irritable

©The miniRQLQ is a coyrighted instrument that may not be altered, sold, translated or adapted for another medium without the permission of Elizabeth Juniper.

Supplementary Note 5*

CONSORT 2010 checklist of information to include when reporting a pilot or feasibility trial[10]

Section/Topic	Item No	Checklist item	Reported on page No
**Title and abstract**			
	1a	Identification as a pilot or feasibility randomised trial in the title	Yes
	1b	Structured summary of pilot trial design, methods, results, and conclusions (for specific guidance see CONSORT abstract extension for pilot trials)	Yes
**Introduction**			
Background and objectives	2a	Scientific background and explanation of rationale for future definitive trial, and reasons for randomised pilot trial	Yes
	2b	Specific objectives or research questions for pilot trial	Yes
**Methods**			
Trial design	3a	Description of pilot trial design (such as parallel, factorial) including allocation ratio	Yes
	3b	Important changes to methods after pilot trial commencement (such as eligibility criteria), with reasons	Yes
Participants	4a	Eligibility criteria for participants	Yes
	4b	Settings and locations where the data were collected	Yes
	4c	How participants were identified and consented	Yes
Interventions	5	The interventions for each group with sufficient details to allow replication, including how and when they were actually administered	Yes
Outcomes	6a	Completely defined prespecified assessments or measurements to address each pilot trial objective specified in 2b, including how and when they were assessed	Yes
	6b	Any changes to pilot trial assessments or measurements after the pilot trial commenced, with reasons	
	6c	If applicable, prespecified criteria used to judge whether, or how, to proceed with future definitive trial	Yes
Sample size	7a	Rationale for numbers in the pilot trial	Yes
	7b	When applicable, explanation of any interim analyses and stopping guidelines	n/a
Randomisation:			
Sequence generation	8a	Method used to generate the random allocation sequence	Yes
	8b	Type of randomisation(s); details of any restriction (such as blocking and block size)	Yes
Allocation concealment mechanism	9	Mechanism used to implement the random allocation sequence (such as sequentially numbered containers), describing any steps taken to conceal the sequence until interventions were assigned	n/a
Implementation	10	Who generated the random allocation sequence, who enrolled participants, and who assigned participants to interventions	Yes

Blinding	11a	If done, who was blinded after assignment to interventions (for example, participants, care providers, those assessing outcomes) and how	n/a
	11b	If relevant, description of the similarity of interventions	n/a
Statistical methods	12	Methods used to address each pilot trial objective whether qualitative or quantitative	Yes
**Results**			
Participant flow (a diagram is strongly recommended)	13a	For each group, the numbers of participants who were approached and/or assessed for eligibility, randomly assigned, received intended treatment, and were assessed for each objective	Yes
	13b	For each group, losses and exclusions after randomisation, together with reasons	Yes
Recruitment	14a	Dates defining the periods of recruitment and follow-up	Yes
	14b	Why the pilot trial ended or was stopped	n/a
Baseline data	15	A table showing baseline demographic and clinical characteristics for each group	Yes
Numbers analysed	16	For each objective, number of participants (denominator) included in each analysis. If relevant, these numbers should be by randomised group	Yes
Outcomes and estimation	17	For each objective, results including expressions of uncertainty (such as 95% confidence interval) for any estimates. If relevant, these results should be by randomised group	Yes
Ancillary analyses	18	Results of any other analyses performed that could be used to inform the future definitive trial	Yes
Harms	19	All important harms or unintended effects in each group (for specific guidance see CONSORT for harms)	n/a
	19a	If relevant, other important unintended consequences	n/a
**Discussion**			
Limitations	20	Pilot trial limitations, addressing sources of potential bias and remaining uncertainty about feasibility	Yes
Generalisability	21	Generalisability (applicability) of pilot trial methods and findings to future definitive trial and other studies	Yes
Interpretation	22	Interpretation consistent with pilot trial objectives and findings, balancing potential benefits and harms, and considering other relevant evidence	Yes
	22a	Implications for progression from pilot to future definitive trial, including any proposed amendments	Yes
**Other information**			
Registration	23	Registration number for pilot trial and name of trial registry	Yes
Protocol	24	Where the pilot trial protocol can be accessed, if available	Yes
Funding	25	Sources of funding and other support (such as supply of drugs), role of funders	Yes
	26	Ethical approval or approval by research review committee, confirmed with reference number	Yes

*We strongly recommend reading this statement in conjunction with the CONSORT 2010, extension to randomised pilot and feasibility trials, Explanation and Elaboration for important clarifications on all the items. If relevant, we also recommend reading CONSORT extensions for cluster randomised trials, non-inferiority and equivalence trials, non-pharmacological treatments, herbal interventions, and pragmatic trials. Additional extensions are forthcoming: for those and for up to date references relevant to this checklist, see www.consort-statement.org.

References:

(1) Kind, P. (1996). The EuroQol Instrument: an index of health-related quality of life. In Spilker, B (Ed). Quality Of Life and Pharmacoeconomics in Clinical Trials. Philadelphia: Lippencott-Raven.

(2) Reilly MC, Zbrozek AS, Dukes EM. The validity and reproducibility of a work productivity and activity impairment instrument. PharmacoEconomics 1993; 4(5):353-65.

(3) Curtis L (2012) *Unit Costs of Health and Social Care 2012*, Personal Social Services Research Unit. The University of Kent. Available at: http://www.pssru.ac.uk/project-pages/unit-costs/2012/ [accessed January 2013]

(4) British National Formulary (January 2013). BMJ Publishing Group and the Royal Pharmaceutical Society of Great Britain. Available at: http://www.bnf.org/bnf/index.htm [accessed January 2013]

(5) Dolan, P., Gudex, C., Kind, P. and Williams, A. (1996) A social tariff for EuroQol: results from a UK general population survey*. Discussion Paper No. 138.*York: Centre for Health Economics, University of York.

(6) 2012 Annual Survey of Hours and Earnings [document on the Internet]. Office for National Statistics; 2012. Available at: http://www.ons.gov.uk/ons/rel/ashe/annual-survey-of-hours-and-earnings/2012-provisional-results/stb-ashe-statistical-bulletin-2012.html [accessed January 2013]

(7) Walters SJ, Brazier JE. Comparison of the minimally important difference for two health state utility measures: EQ-5D and SF-6D. Qual Life Res. 2005; 14(6):1523-32

(8) Porteous, T., Wyke, S., Smith, S., Bond, C., Francis, J., Lee, A. J., et al. 'Help for Hay Fever', a goal-focused intervention for people with intermittent allergic rhinitis, delivered in Scottish community pharmacies: Study protocol for a pilot cluster randomized controlled trial. *Trials* **14:** (2013)

(9) Juniper, E. F., Thompson, A. K., Ferrie, P. J., Roberts, J. N. Development and validation of the mini rhinoconjunctivitis quality of life questionnaire. Clin. Exp. Allergy 30: 132-140 (2000).

(10) Eldridge SM, Chan CL, Campbell MJ, Bond CM, Hopewell S, Thabane L, et al. CONSORT 2010 statement: extension to randomised pilot and feasibility trials. BMJ. 2016;355.
